# Metformin Intervention—A Panacea for Cancer Treatment?

**DOI:** 10.3390/cancers14051336

**Published:** 2022-03-04

**Authors:** Angelika Buczyńska, Iwona Sidorkiewicz, Adam Jacek Krętowski, Monika Zbucka-Krętowska, Agnieszka Adamska

**Affiliations:** 1 Clinical Research Centre, Medical University of Bialystok, 15-276 Bialystok, Poland; iwona.sidorkiewicz@umb.edu.pl (I.S.); adamkretowski@wp.pl (A.J.K.); 2 Department of Endocrinology, Diabetology and Internal Medicine, Medical University of Bialystok, 15-276 Bialystok, Poland; 3 Department of Gynecological Endocrinology and Adolescent Gynecology, Medical University of Bialystok, 15-276 Bialystok, Poland; monikazbucka@wp.pl

**Keywords:** metformin, antioxidant, anti-cancer

## Abstract

**Simple Summary:**

This literature analysis is focused on the pleiotropic action associated with metformin treatment. Precisely, metformin treatment exerts many health effects, mainly by inhibiting inflammatory processes, increasing antioxidant capacity, and improving glycemic and lipid metabolism, which overall may be particularly useful in cancer patients’ clinical management. Consequently, metformin is the main novel therapeutic extensively studied in various clinical trials, which are also summarized in this review.

**Abstract:**

The molecular mechanism of action and the individual influence of various metabolic pathways related to metformin intervention are under current investigation. The available data suggest that metformin provides many advantages, exhibiting anti-inflammatory, anti-cancer, hepatoprotective, cardioprotective, otoprotective, radioprotective, and radio-sensitizing properties depending on cellular context. This literature review was undertaken to provide novel evidence concerning metformin intervention, with a particular emphasis on cancer treatment and prevention. Undoubtedly, the pleiotropic actions associated with metformin include inhibiting inflammatory processes, increasing antioxidant capacity, and improving glycemic and lipid metabolism. Consequently, these characteristics make metformin an attractive medicament to translate to human trials, the promising results of which were also summarized in this review.

## 1. Introduction

Metformin, a biguanide derived from galegine (isoamylene guanidine), has been the main initial pharmacological intervention for type 2 diabetes mellitus (T2DM) since the 20th century [1,2]. Metformin administration has been recommended as the first-line glucose-lowering therapy for all newly diagnosed T2DM by both the American Diabetes Association (ADA) and the European Association for the Study of Diabetes (EASD) since 2009 [1,3]. This medicine is considered to be safe and efficient in monotherapy and in combination with other anti-diabetic therapies [3]. Moreover, it has been shown that adding metformin to insulin therapy to patients with type 1 diabetes mellitus (T1DM) resulted in greater glycemic control and a greater decrease in HbA1c [1].

In addition to decreasing blood sugar levels, metformin is emerging as a therapy with other numerous beneficial effects, including body weight control; reductions in the incidence of aging-related diseases; reductions in the risks of cardiovascular and neuropsychiatric disorders and metabolic syndrome; and even improving fertility in polycystic ovary syndrome (PCOS) [4,5,6,7]. Recent literature data describe metformin as an endothelial protector, an effective drug in heart failure, and an anti-inflammatory target useful in rheumatological and immunological diseases as well as in many aging-related morbidities [8,9,10]. Metformin’s influence on multiple pathways, including lipid metabolism, deactivation of inflammation-related processes, and oxidative homeostasis, has been established [11,12,13,14]. Interestingly, the anti-cancer potential of metformin was firstly recognized in patients with T2DM. Patients treated with metformin were characterized by a lower cancer incidence compared to patients receiving other anti-diabetic therapies [15,16,17]. Moreover, the evidence suggests that metformin treatment is associated with a decreased incidence of certain type of cancers, such as colon, liver, breast, pancreatic, and lung cancer [18,19]. Simultaneous anti-cancer treatment and metformin administration improved the response to cancer therapy and decreased cancer mortality [15,20]. With the wide pleiotropic effect of metformin, only 20% of side effects occur, mainly in the gastrointestinal tract. Therefore, this therapy could be used in most clinical management protocols. Moreover, metformin is the main novel therapeutic focus of various clinical trials [21].

This literature review was undertaken to summarize novel evidence concerning metformin intervention with a particular emphasis on its antioxidant and anti-cancer properties. Furthermore, the promising results obtained in many clinical trials concerning metformin intervention are also presented in this review [15,22,23,24,25,26]. 

## 2. Multifactorial Actions of Metformin

Metformin is generally known for its anti-diabetic effects. Approximately 40–60% of orally administered metformin is absorbed into the blood. The maximum blood levels are reached after approximately 2.5 h for the immediate-release and 7 h for the extended-release tablet forms [27]. Following the oral ingestion of metformin, this therapeutic is absorbed by enterocytes through the plasma monoamine transporter (PMAT; encoded by the gene Solute Carrier Family 29 Member 4 (SLC29A4), and the organic cation transporter 3 (OCT3), encoded by the gene Solute Carrier Family 22 Member 3, SCL22A3) [28,29]. In hepatocytes, the organic cation transporter 1 (OCT1) and OCT3 are responsible for metformin absorption [30]. Metformin is extracted into the bile by multidrug toxin and extrusion 1 (MATE1) (encoded by Solute Carrier Family 47 Member 1, SLC47A1) [31]. Then, metformin metabolites are excreted unchanged into the urine, where the organic cation transporter 2 (OCT2) (encoded by Solute Carrier Family 22 Member 2, SLC22A2) transports it into the renal epithelial cells through the basolateral membrane, and MATE1 and MATE2 (encoded by Solute Carrier Family 47 Member 2, SLC47A2) excrete it into the urine [31,32,33,34]. 

Metformin is able to directly and indirectly interact with many enzymes, such as mitochondrial electron transport chain (ETC) complex I, adenosine 5′-monophosphate-activated protein kinase (AMPK), glycerol 3-phosphate dehydrogenase, fructose 1-6-bisphosphatase, and glucose 6P-phosphatase, which highlights the metabolic properties of metformin [15,35,36,37,38,39,40]. Metformin’s influence on multiple metabolic pathways is produced by targeting ETC complex I, the inhibition of which leads to subsequent AMPK activation [41,42,43]. AMPK is the most significant regulator of many metabolic pathways related to lipid metabolism, insulin sensitivity, and energy homeostasis [6,15,27,44]. Nevertheless, several other metabolic properties should be also considered as a major contributor to the therapeutic action of metformin, particularly inhibition of fructose 1-6-bisphosphatase, a gluconeogenesis rate-controlling enzyme, via lowering hepatic glucose production [45]. Moreover, metformin decreases glucose 6-phosphatase activity in hepatocytes by activation of glycolysis downstream of glucose phosphorylation [46]. These mechanisms are implicated in the subsequent pentose-phosphate pathway in the endoplasmic reticulum, constituting the principal component involved in cellular proliferation and antioxidant processes [47]. 

The reduction in insulin resistance after metformin treatment is associated with an increase in plasma insulin-like growth factor-binding protein 1 (IGFBP-1) concentrations and a decrease in the insulin-like growth factor (IGF-I)/IGFBP-1 ratio [48,49]. This biguanide is also implicated in the redistribution of glucose transporters—glucose transporter 1 (GLUT-1) and glucose transporter 4 (GLUT-4)—from the intracellular space to the cell membranes [50,51]. One of metformin’s mechanisms of action is the activation of insulin receptor substrate 2 (IRS-2) in the liver, and the downstream increase in deoxyglucose uptake is mediated via increased translocation of GLUT-1 to the plasma membrane [52]. 

Importantly, metformin administration is also associated with the regulation of many molecular targets, such as mitogen-activated protein kinases (MAPKs), B-cell CLL (BCL-2), signal transducer and activator of transcription 3 (STAT3), glioma-associated oncogene homolog 1 (GLI1), mitogen-activated protein kinase 1/2 (ERK1/2), and ribosomal S6 kinase (RSK1) [14,53,54,55,56,57,58]. Using gene expression analysis after metformin intervention, the expression of key genes and their associated proteins was assessed to evaluate metformin’s anti-cancer properties (Table 1).

Nevertheless, metformin exerts hermetic effects [68]. The metformin dose–response action is characterized by a low dose stimulation and high dose inhibition [69]. This phenomenon can be explained using an example of the activation of mitochondrial complex I by metformin administration [41]. In in vivo studies, the inhibition of respiratory complex I was achieved after 10 mM or 30 mmol/L metformin treatment [70,71,72]. On the other hand, a study performed by Larsen et al. revealed that a high dose of metformin does not impact mitochondrial complex I respiration in skeletal muscle of patients with T2DM [73]. The inhibition of respiratory complex I and AMPK phosphorylation is achieved using concentrations higher than 1 mM, which is above the maximum therapeutic doses used (generally, 500–2500 mg/day). In contrast, therapeutic concentrations of metformin induce activation of mitochondrial energy metabolism with an improvement in cellular energy status [74]. The concept of hormesis can be useful to fully understand the effective tailoring of metformin therapy [69,75,76].

## 3. Metformin as a Promoter of Antioxidant Defense

Oxidative stress is an imbalance between the production of reactive oxygen species (ROS) and the organism system’s ability to detoxify and may cause a toxic effects through the production of lipid peroxides and ROS, which could result in damage of components, such as proteins, lipids, and DNA [77]. These damages are mostly caused by O^2−^ (superoxide radical), OH– (hydroxyl radical), and H_2_O_2_ (hydrogen peroxide) [78,79,80].

While multiple reports based on in vivo and in vitro studies provide evidence that oxidative-stress markers unequivocally decrease in metformin-treated individuals, the details of the mechanisms responsible for these changes are not thoroughly understood [81,82,83,84,85,86]. The in vitro study performed by Javadipour et al., which focused on the antioxidant effects associated with metformin, revealed that metformin protected against increased oxidative stress via the nicotinamide adenine dinucleotide (NAD)-dependent protein deacylase sirtuin-3 (*SIRT3*)-related pathway [86,87]. The study was conducted on mitochondria isolated from rat pancreases, and increased oxidative stress and insulin resistance were induced through arsenic exposure [86]. Additionally, metformin is a direct Sirtuin 1 (SIRT1) activating compound, which is a crucial cellular survival protein, thus also involved in combatting oxidative stress [88,89]. Experimental studies indicate that SIRT1 may play a role in the pathogenesis of skeletal muscle insulin sensitivity. SIRT1 directly influences the insulin signal transduction pathway. It increases insulin-dependent IRS2 phosphorylation and Akt activation. Moreover, SIRT1 interacts with peroxisome proliferator-activated receptor α coactivator 1 (PGC1α) and AMPK to stimulate muscle glucose uptake and fatty acid oxidation, and thus it can prevent insulin resistance. Therefore, SIRT1 activators might be useful in the treatment of insulin resistance-related diseases [90].

Accordingly, in the following studies, the metformin-dependent mechanisms involved in enhancing the endogenous antioxidant system were also evaluated. Literature data reported the upregulation of the activity of antioxidant enzymes, including glutathione reductase, catalase, and superoxide dismutase (SOD), and GSH through the downregulation of nicotinamide adenine dinucleotide phosphate (NADPH) oxidase [82,91,92,93]. Moreover, metformin’s modulation of oxidative phosphorylation and glycolysis is mediated by mitochondrial-derived glycerophosphate dehydrogenase (MGPDH) by significant MGPDH downregulation [20]. In addition, metformin can reduce oxidative stress and promote autophagic processes through the activation of AMPK and SIRT3 [53,85,94]. Moreover, paraoxonase 1 (PON1) is the antioxidant that circulates in association with high-density lipoprotein (HDL), simultaneously eliminating the lipid peroxides within lipoproteins [95,96,97]. It is considered to be an HDL lipid-oxidation protector. The study, which was performed using diabetic animal models [54,85] and patients with newly diagnosed T2DM [98] treated with metformin, proved that after this drug intervention, the activity of PON1 was increased (Table 2). 

In addition, an in vivo animal study suggested that metformin may protect against ROS [83]. Accordingly, the administration of metformin increases the survival of cells exposed to 4–7 Gray of radiation [100]. An additional advantage is a protective effect 24 h after exposure to radiation, which is potentially useful in counteracting the previously received radiation [100,101]. These reports emphasize the necessity of continuing to evaluate metformin-induced antioxidant mechanisms, therefore, to provide protection against ROS and radiation-induced oxidative damage. 

## 4. Anti-Cancer Effect

There is growing interest in the potential benefits of metformin in cancer treatment and prevention [60]. Tumorigenesis is a complex relationship of metabolic processes that requires a number of steps, in which hyperglycemia may be the main modulator. Cancer cells require high glucose uptake for unrestrained cell growth [6,21,102]. Metformin treatment may not only reduce the risk of cancer patient mortality but may also improve the efficacy of cancer treatment [22,103]. A study performed on patients with T2DM treated with metformin revealed that cancer incidence and mortality were decreased by approximately 10% to 40% compared to that in patients not treated with metformin [14,104]. 

Mallik et al. showed that AMPK, which is a regulator of multiple processes in cells, specifically acts to inhibit cellular proliferation by disrupting cell division in mitosis, thus playing an essential role in cancer pathogenesis [105]. The increase in AMPK activity induced by metformin appears to induce the G1 phase of the cell cycle, which leads to the arrest of cell proliferation without activating the apoptotic mechanism [42]. Moreover, through AMPK activation, metformin indirectly reduces cancer cell viability by inducing apoptosis and inhibits osteoclast differentiation in bone tumors [106,107]. Furthermore, the reduction in blood glucose and insulin levels may impair the cancer’s growth by AMPK activation, which leads to decreased transcription of gluconeogenic genes by inhibition of histone deacetylases (HDAC) and decreased lipogenic gene expression by inhibition of sterol regulatory element binding proteins [108]. 

The mammalian target of rapamycin (mTOR) is one of the major intracellular factors involved in modulating cell growth, proliferation, and metabolism [109,110,111]. The overexpression of mTOR is often associated with tumor progression, diabetes, and neurodegenerative disorders [19,105]. It has been shown that metformin inhibits mTOR activity, and this may help to inhibit the multiplication of neoplastic cells [37,112,113]. Moreover, mTOR kinase integrates cell-signaling pathways, including the insulin pathway, and growth factors such as insulin-like growth factor 1 (IGF-1) and 2 (IGF-2), and acts as a sensor for the cellular levels of energetic compounds [62]. Typically, under aerobic conditions, energy is produced through glycolysis followed by the citric acid cycle and oxidative phosphorylation [114]. However, the pathway of energy production in cancer cells differs; the citric acid cycle and oxidative phosphorylation are limited, even under aerobic conditions, to favor lactic fermentation, which is called the “Warburg effect”. This process produces significantly less ATP, which leads to increased energy uptake and use of nutrients, such as nucleotides, amino acids, and lipids, which in turn favor the proliferation of cancer cells [115]. Importantly, metformin, by reducing hyperglycemia and normalizing lipid metabolism, inhibits the Warburg effect, thereby hindering cancer proliferation. 

Protein 53 (p53) is a tumor suppressor that regulates autophagic and apoptotic processes [116]. Accumulating evidence suggests that p53 activation can transcriptionally inhibit GLUT1 and GLUT4 expression, suppressing glucose uptake [117]. Moreover, p53 induces the expression of TP53-induced glycolysis and apoptosis regulator (TIGAR), a transcription factor involved in the regulation of ROS formation, autophagy, and apoptosis [118]. Accordingly, metformin’s anti-cancer effect could be related to the modulation of AMPK, which leads to subsequent p53 activation [105,116]. In one study, the reduced stability of p53 in metformin-treated individuals led to a decrease in differentiated embryo chondrocyte 1 (DEC1) expression, which plays a crucial role in the DNA-damage response via transcriptional regulation under hypoxic conditions and the induction of cancer cell apoptosis [19,21,119].

It has been shown that the inhibition of epidermal growth factor receptor 2 (HER2) expression leads to reduced expression of vascular endothelial growth factor A (VEGFA), ultimately resulting in lower angiogenesis and anti-cancer effects [120]. Interestingly, Wang et al. reported that metformin could reduce the expression of HER2 [121]. Furthermore, metformin has been shown to reduce the synthesis of lithocholic acid (LCA), which is additionally stimulated by increased oxidative stress [122], leading to an inhibitory effect on NF-κB signaling. NF-κB signaling is essential for the regulation of interleukin 8 (IL-8), which plays an important role in tumor progression and metastasis in a variety of human cancers, especially in lung and colon cancers [123,124]. In addition, metformin inhibits hexokinase (HK) in breast cancer cells, which is an essential glycolytic enzyme that catalyzes the phosphorylation of glucose by ATP to glucose-6-phosphate (G6P). It has been shown that G6P partially impairs glucose metabolism and tumor growth [125]. The study performed by Bonannii et al. showed that in a group of diabetic patients also diagnosed with breast cancer treated with metformin, the tumor size was reduced compared to that in the control group [126]. The trend of decreasing breast cancer proliferation in the diabetes patient group suggests that indirect insulin- and glucose-mediated effects are the main mechanism of anticancer effect of metformin in human breast cancer and attenuate the role of direct effects on specific pathways concerned on the AMPK/mTOR cascade activity [126]. The prognosis of neoplastic disease development can be determined based on the expression of genes encoding angiogenic factors. Research focused on angiogenesis is justified in order to identify useful prognostic factors, find diagnostic markers, and determine new therapeutic targets for introducing potential new-generation medicines. 

What should also be noted in the context of the anti-cancer action of metformin is that this therapeutic inhibits the production of leptin and stimulates the production of adiponectin [127]. Increased serum leptin levels have been shown to be associated with increased tumor growth and a greater risk of metastasis. By contrast, adiponectin may present an inhibitory effect on cancer development and appears to exert an anti-proliferative effect in tumor cells. 

Overall, the results demonstrate that metformin acts comprehensively on various cancers, such as breast, pancreatic, gastric, colorectal, endometrial, prostate, and bladder cancer, and could represent an independent treatment option or be used in combination with other medicines [18,19,22,98,128,129,130]. In many studies performed in a group of lung cancer patients treated with metformin, decreased proliferation of cancer cells and increased apoptosis were reported [102,131,132,133,134]. Growing evidence from epidemiological and observational studies shows that metformin may be an effective adjuvant anti-cancer therapy [24,60,132].

## 5. Pleiotropic Use of Metformin in Clinical Trials

### 5.1. Metabolic Effect of Metformin in Various Diseases

Currently, 2035 clinical trials using metformin are registered as completed or ongoing according to the Protocol Registration and Results System (PRS) and the European Union Clinical Trials Register. This underlines the growing interest in this therapy [135]. Numerous clinical studies have demonstrated that metformin monotherapy is effective in reducing the risk of serious complications associated with T2DM with a median dose of 1500–2000 mg/day [136,137,138,139]. The beneficial effects of metformin in reducing the risk factors of atherosclerosis, improving microcirculatory blood flow, and, in particular, increasing the sensitivity of cells to insulin are well documented [140]. Metformin also improves the function of the heart muscle cells. Increasing glucose metabolism along the glycolysis pathway through the inhibition of fatty acid β-oxidation reduces the oxygen demand of myocardial cells. This has a positive effect on the efficiency of the Na/K/Ca membrane pumps [141,142]. The increased removal of Ca^2+^ ions from the cytoplasm of cardiomyocytes improves the relaxation of the heart muscle [143]. Additionally, metformin intervention in T2DM patients also resulted in a reduction in the risk of Parkinson’s disease, dementia, and other neurodegenerative diseases [144,145]. The study performed by Hsu et al. reported that T2DM increased the risk of dementia 2.6-fold, and the combined intervention of sulfonylureas and metformin was able to decrease the risk of dementia by 35% in an 8-year observation [145]. 

It has been shown that obesity is associated with increased risk of the cancer development, whereas metformin treatment is associated with weight reduction [146,147,148,149]. The clinical trial conducted by Ruholamin et al., in a group of women diagnosed with gestational diabetes who received 500 mg metformin once or twice a day, demonstrated that metformin intervention resulted in weight and insulin dose reductions, leading to better glycemic control. Moreover, newborns were observed to have a decreased rate of obesity [150,151,152]. The study conducted by Seifarth et al., which focused on the effect of metformin intervention on weight loss in non-diabetic obese individuals, suggested that this treatment option was an effective tool for weight reduction in both insulin-sensitive and insulin-resistant overweight patients and obese patients [26]. This study included 154 patients with body mass indices ≥27 kg/m^2^ who received metformin intervention dosages of 2500 mg per day in an outpatient setting over 6 months [26].

### 5.2. Effect of Metformin on Fertility

There are currently 349 female infertility trials, many of which are focused on the increased efficacy of in vitro fertilization (IVF) when combined with metformin intervention [153]. The clinical trial performed by Foda et al. showed that metformin therapy in patients with endometriosis resulted in an increased pregnancy rate and lower serum cytokine levels [154]. For the purpose of this study, 35 infertile patients with endometriosis were administered with 500 mg metformin three times daily for 6 months plus a multivitamin once daily. A literature review on metformin intervention in the treatment of infertility in PCOS revealed 10 completed clinical trials [153]. The studies demonstrated that metformin was effective in inducing ovulation and reducing the risk of preterm labor in PCOS patients. A comprehensive review of seven randomized clinical trials involving 702 women unsuccessfully trying to become pregnant for a period of 6 months showed an increased clinical pregnancy rate after metformin treatment. Moreover, these studies confirmed that metformin therapy improved blood supply and the thickness of the endometrium in PCOS women [155]. Additionally, Johnson suggested that women with PCOS undergoing IVF should be treated with metformin to reduce the risk of ovarian hyperstimulation syndrome [156]. In 2019, a promising metformin intervention in a group of women with unexplained infertility with anovulatory cycles was begun (NCT03681197) [157].

### 5.3. Anti-Cancer Effect of Metformin in Cancer in Clinical Trials

Currently, 255 clinical trials concerning the potential use of metformin in cancer treatment are being conducted. There is strong evidence concerning the association between metformin use and decreased pancreatic cancer incidence and increased overall survival in colorectal cancer [158,159]. The study conducted by Miranda et al. on patients with colorectal cancer revealed that 850 mg of metformin, 425 mg/m^2^ 5-fluorouracil, and 50 mg leucovorin twice a day was associated with longer survival in obese patients [160]. In another METAL (metformin in advanced lung cancer) trial, the clinical utility of metformin with erlotinib in second-line therapy of patients with stage IV non-small-cell lung cancer was evaluated. For the purpose of this study, twelve patients were enrolled and administered with 1500 mg metformin with 150 mg erlotinib. The study showed that this combination improved prognoses for patients. This approach could also improve survival and overall outcome [161]. The clinical trial conducted by Bever et al., in which 22 patients with metastatic pancreatic adenocarcinoma were treated with metformin alone (850 mg orally twice a day) or with rapamycin (4 mg daily), showed that the intervention was well tolerated, and that certain patients achieved stable disease, which was further associated with longer survival [162]. Moreover, interim analyses of ongoing studies involving neoadjuvant metformin treatment in newly diagnosed breast cancer patients demonstrate that this intervention is safe and well tolerated. It was proved that metformin can directly affect primary breast cancer in vivo, including the downregulation of phosphodiesterase 3B *(PDE3B)*, a critical regulator of cAMP synthesis, which combined with AMPK activation, can be considered as adjuvant breast cancer therapy [163]. The clinical trial conducted by Goodwin et al. suggested that the administration of 850 mg of metformin led to weight loss and improved metabolic health in early-stage breast cancer patients. Furthermore, support for a potential metformin treatment mediated by reducing serum levels of insulin and other metabolic markers, such as serum levels of glucose, leptin, and high-sensitivity C-reactive protein (hsCRP) in a group of breast cancer patients was provided [164]. Patients receive oral metformin hydrochloride twice a day (once daily in weeks 1–4). Treatment continues for up to 5 years in receptor positive (estrogen receptor and/or progesterone receptor positive) subjects in the absence of disease progression or unacceptable toxicity. Contrarily, the clinical trial of Zhao et al. concerning aromatase-inhibitor treatment combined with 500 mg metformin orally in pre-treated postmenopausal patients with hormone receptor-positive metastatic breast cancer returned negative results although with excellent tolerability [109]. However, the study performed by Monami et al., in which 195 patients were included over a period of 9.6 years, proved that metformin intervention for more than 36 months was associated with a significant reduction in the risk of cancer [165]. According to a cohort study, patients with esophageal cancer (in a study group of 285 patients) and patients with rectal cancer (in a study group of 472 patients) receiving a combination of metformin with radiotherapy/chemotherapy demonstrated increased responses to the anti-cancer treatment and improved prognoses [24].

## 6. Can Metformin Intervention Be Considered as Adjuvant Anti-Cancer Therapy?

### 6.1. Anti-Cancer Effect of Metformin in Thyroid Cancer

In recent years, the global incidence rates of thyroid cancer (TC) have been steadily rising [15]. Recent reports indicate that metformin may exert anti-tumor activity by inhibiting tumor cell proliferation and inducing apoptosis [62,125,166]. It has been documented that TC diabetic patients treated with metformin are characterized by smaller tumor sizes, higher complete remission rates, and longer progression-free survival compared to diabetic patients not treated with metformin [4]. Ye et al. described the in vitro effects of 10 and 20 mM of metformin on human papillary TC using a human papillary TC cell line (TPC-1). The use of metformin increased the expression of several factors, including the heat shock 70-kDa protein 5 (Hspa5), also known as binding immunoglobulin protein (BIP), C/EBP homologous protein (CHOP), and caspase-12, which activate endoplasmic reticulum (ER) stress conditions, leading to cancer cell apoptosis [55,64]. Using flow cytometry, a significant increase in TPC-1 cell apoptosis after metformin intervention was observed as compared to in the control group. The study performed using in vitro and in vivo models concluded that metformin can promote apoptosis through endoplasmic-reticulum-stress-associated pathways following the increased expression of BiP, DNA damage-inducible transcript 3, and caspase-12 [167]. Han et al. suggested that metformin inhibits TC cell growth, migration, and the epithelial-to-mesenchymal transition by inhibiting the mTOR pathway, where the metformin treatment was given at a concentration of 10 mM concentration [168]. The study performed by Thakur et al. demonstrated that MGPDH regulated human TC cell growth and oxidative phosphorylation (OXPHOS) [20]. Moreover, MGPDH overexpression was associated with an increase in thyroid cell proliferation. Interestingly, downregulation of MGPDH expression and OXPHOS inhibition in TC in vitro was observed after 48 h 5 mM metformin treatment [20]. Using TC cells (FTC133 and BCPAP), Bikas et al. demonstrated that the combination of metformin and other glycolysis inhibitors improved upon current TC treatments. Moreover, due to providing the decrease in cellular ATP, the prolonged activation of AMPK, and the sustained autophagy, adding metformin to already used treatment regimens resulted in a broader cancer-treatment spectrum [55,107].

Radioactive iodine therapy (RAI) is the standard treatment for differentiated thyroid cancer (DTC). The increased oxidative stress, reflected by malondialdehyde (MDA) measurements, further enhanced by RAI in DTC patients may have important implications for future health complications [169,170]. Despite the beneficial therapeutic effect of RAI intervention, exposure to radiation can lead to several oxidative alterations, especially in the metabolism of reproductive system tissue [7,171]. Over the last decade, evidence of the anti-cancer effects of metformin—the most widely prescribed anti-diabetic medicine in the world—shows its usefulness in the clinical management of endocrine malignancies [15,21,61,172,173,174]. 

### 6.2. Metformin Intervention in Endometrial Cancer

Endometrial cancer (EC) is the most common gynecological malignancy and is characterized by hypermenorrhea, dysfunctional uterine bleeding, and infertility [175,176]. The current recommendations from the Cancer Genome Atlas (TCGA) define four clinically distinct endometrial cancer types based on their p53 mutational burden, the copy number variations, the exonuclease domain of the DNA polymerase epsilon (POLE) mutations, and the microsatellite instability [177]. Furthermore, EC is usually associated with PCOS, obesity, insulin resistance, and T2DM [156,178]. The impact of insulin and IGF-1 expression have an important role in the development of EC. Furthermore, the aggressiveness of EC has been shown to correspond with elevated levels of circulating insulin and endometrial IGF-1 concentrations. Another potentially important element in the mechanism through which metformin inhibits the development of EC is related to increased GLUT4 activity, which is combined with cell proliferation inhibition and cell cycle arrest and apoptosis induction [178]. Moreover, the data demonstrate that female sex steroids regulate tissue insulin sensitivity and modulate the intracellular glucose pathways [7,179,180]. In this regard, metformin increases the blood levels of sex hormone binding globulin, which leads to a reduction in the circulating estrogen and androgen concentrations [178,181]. It has been reported that 2 mM of metformin improved EC hormonal treatment by causing a regression to histologically normal endometrium, enhancing healing, and reducing any side effects [175,178,182]. Additionally, 500 mg of metformin orally, three times a day, is capable of overcoming progestin resistance in endometrial cancer cells [183]. It has been suggested that metformin’s anti-cancer properties result from its ability to alter glucose metabolism, activating AMPK and inhibiting the PI3K/AKT/mTOR signaling pathway [184,185]. In particular, numerous studies have examined the effectiveness of targeted therapies acting on the PI3K/AKT/mTOR pathway, epidermal growth factor receptor (EGFR), human epidermal growth factor receptor 2 (HER2), and vascular endothelial growth factor (VEGF), which have been shown to be metformin targets [178,186,187]. The use of targeted therapies appears to be key to achieving better responses and survival among women with advanced or recurrent EC [188]. In this regard, the pleiotropic effects of metformin on cellular energy production with intercellular and hormone-based interactions make metformin a potential anti-cancer treatment for EC.

There are 14 clinical trials being conducted to assess the effectiveness of metformin medication in the treatment of endometrial hyperplasia and cancer [189]. Studies of the therapeutic properties of metformin in EC patients have been published, showing that metformin treatment functions as an useful preventive therapy for neoplastic diseases [190,191]. The results from clinical trials suggest that metformin intervention (750–2250 mg/day administered for 24–36 weeks to achieve a complete response) combined with medroxyprogesterone acetate (MPA) improves the regression of cancer cells, providing additional protective effects on fertility in EC patients [192]. Current clinical trials showed that progestin fertility-sparing treatment combined with 500–1500 mg daily of metformin shortened the treatment time, reducing the risk of side effects and endometrial damage during the treatment of EC [178,181,183,189,190,191,193]. 

## 7. Materials and Methods

**Figure 1:**
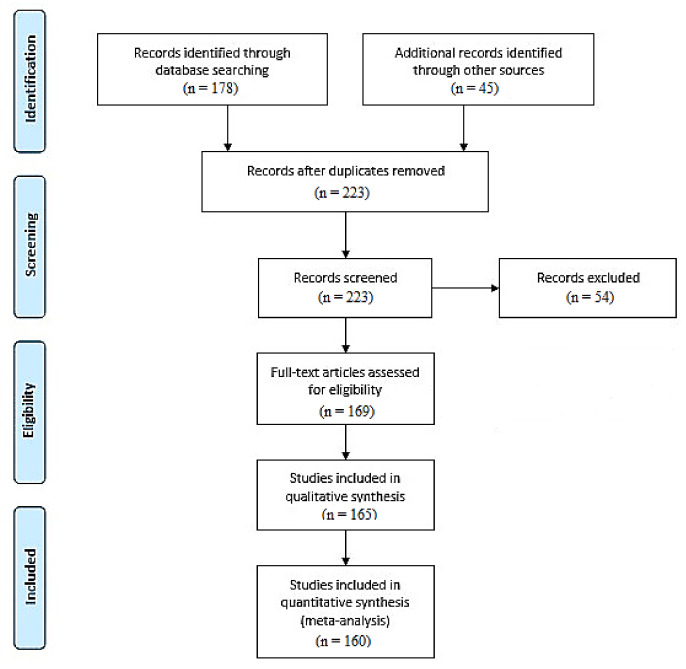
PRISMA-based flow diagram of the meta-analysis process performed [194,198].

The literature review was performed using the PubMed database and according to the PRISMA and EQUATOR network guidelines [194,195,196,197]. For the purpose of this review, medical papers published in 2004–2021 were analyzed. Systematic review of the current literature about the metformin intervention was performed. The keywords used in the literature search were as follows: metformin, metformin pharmacokinetics, anti-cancer therapy, antioxidant therapy, metformin therapy, potential therapy, clinical trials, thyroid cancer, and endometrial cancer. Studies evaluating the latest reports based on anticancer properties, the impact of oxidative stress, and potential therapeutic targets related to metformin intervention were included. Articles with irrelevant conclusion statements, inappropriate study methods, inadequate reporting, or incomplete reports were excluded from the study (Figure 1). This review has been registered in PROSPERO (CRD42022299568).

## 8. Conclusions

Undoubtedly, metformin exerts pleiotropic effects on many metabolic pathways. One of metformin’s most significant potential applications is cancer treatment. Studies using in vitro models focused on metformin’s anti-cancer mechanisms and potential uses produced favorable results. Therefore, based on this evidence, metformin can be used widely in relation to thyroid, endometrial, breast, pancreas, and lung cancers, which are epidemic in modern societies according to the reputably published literature data. However, further randomized clinical trials to assess metformin’s individual metabolic effects and specific molecular mechanisms are warranted. There is a high probability that introduction of metformin to therapeutic protocols could extend the period of cancer non-recurrence and reduce the side effects of chemotherapy and radiotherapy. Thus, the development of novel indications for this therapy is still required. 

## Figures and Tables

**Table 1:** Metformin’s metabolic targets.

Gene/Protein Abbreviation	Full Name	Form of Dysregulation	Reference
MAPK	Mitogen-activated protein kinase	Down	[59]
BCL-2	B-cell CLL/lymphoma 2	Down	[60]
P38	Mitogen-activated protein kinase p38	Down	[59,61]
JNK	c-Jun N-terminal kinase	Down	[59]
STAT3	Signal transducer and activator of transcription 3	Down	[62]
GLI1	Glioma-associated oncogene homolog 1	Down	[63]
MEK1/2	Dual specificity mitogen-activated protein kinase ½	Up	[63]
TP	Thymidine phosphorylase	Down	[64]
ERCC1	Excision repair cross-complementation 1	Down	[64]
ERK1/2	Mitogen-activated protein kinase ½	Up	[65]
RSK1	Ribosomal S6 kinase	Up	[66]
AMPK	AMP-activated protein kinase	Up	[66]
mTOR	Mammalian target of rapamycin	Down	[65]
AKT	RAC-alpha serine/threonine-protein kinase	Down	[65]
P27	Cyclin-dependent kinase inhibitor 1B	Up	[67]

**Table 2:** Metformin’s antioxidant targets.

Protein Name	Full Name	Form of Dysregulation	Reference
GR	Glutathione reductase	Down	[36]
MGPDH	Mitochondrial glycerophosphate dehydrogenase	Down	[20]
NADPH oxidase	Nicotinamide adenine dinucleotide phosphate oxidase	Down	[82]
PON1	Paraoxonase 1	Up	[95]
SOD	Superoxide dismutase	Down	[36]
SIRT1	Sirtuin 1	Up	[85,99]
